# Concomitant DNA methylation and transcriptome signatures define epidermal responses to acute solar UV radiation

**DOI:** 10.1038/s41598-020-69683-8

**Published:** 2020-07-31

**Authors:** Nicholas Holzscheck, Jörn Söhle, Torsten Schläger, Cassandra Falckenhayn, Elke Grönniger, Ludger Kolbe, Horst Wenck, Lara Terstegen, Lars Kaderali, Marc Winnefeld, Katharina Gorges

**Affiliations:** 1 Beiersdorf AG, Research and Development, Troplowitzstraße 17, 22529 Hamburg, Germany; 2 Institute for Bioinformatics, University Medicine Greifswald, Greifswald, Germany

**Keywords:** Computational biology and bioinformatics, Genetics, DNA methylation, Molecular biology, Transcriptomics

## Abstract

The simultaneous analysis of different regulatory levels of biological phenomena by means of multi-omics data integration has proven an invaluable tool in modern precision medicine, yet many processes ultimately paving the way towards disease manifestation remain elusive and have not been studied in this regard. Here we investigated the early molecular events following repetitive UV irradiation of in vivo healthy human skin in depth on transcriptomic and epigenetic level. Our results provide first hints towards an immediate acquisition of epigenetic memories related to aging and cancer and demonstrate significantly correlated epigenetic and transcriptomic responses to irradiation stress. The data allowed the precise prediction of inter-individual UV sensitivity, and molecular subtyping on the integrated post-irradiation multi-omics data established the existence of three latent molecular phototypes. Importantly, further analysis suggested a form of melanin-independent DNA damage protection in subjects with higher innate UV resilience. This work establishes a high-resolution molecular landscape of the acute epidermal UV response and demonstrates the potential of integrative analyses to untangle complex and heterogeneous biological responses.

## Introduction

Solar UV irradiation has complex and ambivalent effects on the human organism. Beneficial effects of sun exposure are thought to be mainly mediated by vitamin D, which is synthesized in the skin through a photosynthetic reaction triggered by exposure to UVB. Vitamin D was primarily acknowledged for its importance in bone formation, increasing evidence however points to its influence on the proper functioning of nearly every tissue in our bodies^1^. In contrast to this however, solar UV irradiation is also the most abundant risk factor for skin cancer and other extrinsically influenced skin disorders^2,3^. It is well established that UV irradiation both directly and indirectly induces DNA damage. Direct damage is mainly a result of UVB and to lesser extent UVA irradiation, causing dimerization of adjacent pyrimidine bases, a frequent cause of mutations during replication^4^. Indirect DNA damage results mainly from oxidative stress, caused by free radicals and cellular reactive oxygen species, which increase after UV irradiation^5^. Damaged DNA, if not properly repaired, interferes with many cellular mechanisms such as transcription, the cell cycle and replication and can give rise to mutations and epigenetic alterations, driving genomic instability and ultimately carcinogenesis.

Human skin has developed several defense systems to guard against the damaging effects of UV: Prominently these include structural changes to the tissue such as epidermal thickening and the synthesis of melanin, but they also comprise quick molecular adaptations like the suspension of cell cycle and gene transcription, as well as the activation of DNA repair pathways. The extent of protection afforded by these mechanisms however is characterized by high inter-individual variation^6^. The stratification of individual UV response is thus highly important for risk assessment in cancer prevention (UV-protection), therapeutic dose determination (PUVA therapy) and in the understanding of the biological processes leading to malignancies (e.g. squamous skin cancers). Fitzpatrick skin type categories^7^ have been widely used as an indicator and predictor of sun sensitivity in epidemiology and experimental photobiology. However, this categorization is hampered by subjectivity and is prone to recall error^8^. In a study assessing the reliability of Fitzpatrick skin type classifications for instance, only ~ 60% of all study participants self‐identified as the same skin type after repeated questioning a few months later^9^. Several authors have investigated the relationship between Fitzpatrick skin type and minimal erythema dose (MED)^10–13^, a more objective measure of UV sensitivity frequently used in clinical or research settings, showing increasing MED with higher Fitzpatrick classification in general, but with considerable intergroup variation.

DNA methylation is a covalent epigenetic modification of cytosine to 5-methylcytosine, occurring within CpG dinucleotides^14,15^. Although methylations of adenine have been reported as well, these have so far received considerably less attention. Methylation of CpG sites in the human genome is an important regulatory mechanism that can lead to the activation or repression of gene transcription. Modifications are established and maintained by a set of specific enzymes called DNA methyltransferases. DNA methylation is generally considered to represent a regulatory interface between environmental cues and the genome and might cause or allow long-lasting changes in gene transcriptional activity^16^. Our current knowledge about epigenetic changes associated with acute UV irradiation, its contribution to transcriptomic alterations and implication in skin photobiology, remains very limited. Previous studies have shown however, that chronic solar UV gives rise to large hypomethylated blocks of DNA in the healthy epidermis and that these blocks are conserved in cutaneous squamous skin carcinomas^17^, underlining the importance of studying DNA methylation in the context of solar irradiation. In addition, a multi-omics analysis of UV irradiated keratinocytes recently identified several new UV target genes including CYP24A1, GJA5, SLAMF7 and ETV1^18^, demonstrating the value of multi-layered omics analyses in unraveling biological phenomena and enabling more reliable biomarker detection, as it has similarly been shown in cancer research, allowing molecular diagnosis and prognosis, often utilizing DNA methylation markers^19,20^.

Here we hypothesized that integrative analysis of UV induced epigenetic and transcriptomic alterations in vivo might help to decipher inter-individual responses to environmental challenges and give hints towards early pathogenesis. For this reason, we generated high-resolution multi-omics molecular profiles of the in vivo irradiated epidermis. Our results provide evidence that a UV induced epigenetic memory might be established already after short term repetitive UV irradiation. Integrative analyses of methylation and expression data reveal previously unnoted pathways involved in the acute epidermal UV response and allow the precise inter-individual prediction of MED without the need for prior UV irradiation. Finally, analysis of these molecular phototypes indicates the existence of a melanin-independent form of damage protection in individuals with higher innate resilience to UV irradiation.

## Results

### UV irradiated epidermis shows genome-wide aberrant methylation patterns and substantial transcriptomic reprogramming

**Figure 1:**
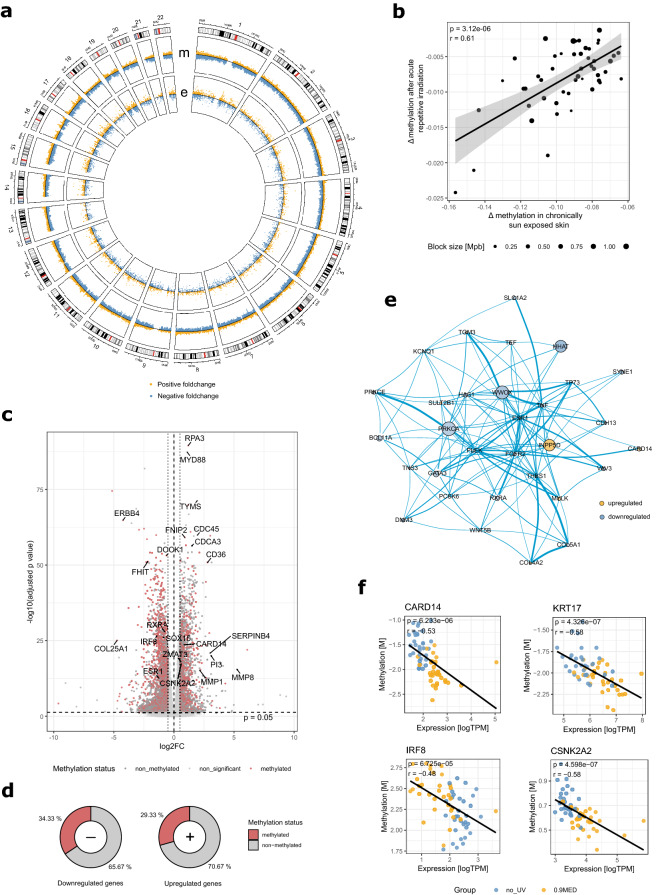
Epigenetic and transcriptomic changes of irradiated samples compared to non-irradiated controls: (**a**) Circos plot showing differential methylation (m, outer circle) and expression (e, inner circle) in response to irradiation to 0.9 MED in a genomic context (FDR < 0.05). Amplitude of points corresponds to log2 fold-change with the solid black line representing no change. Hypomethylated CpGs and downregulated genes are colored in blue, hypermethylated CpGs and upregulated genes in yellow. Colored bands in the karyogram mark centromeres (red) and heterochromatin status (grey to black). (**b**) Differential methylation of 49 genomic regions previously associated with chronic sun-exposure^17^ compared to differential methylation after acute repetitive irradiation. (**c**) Volcano plot of differential gene expression in response to irradiation. Differentially expressed genes with ≥ 3 differentially methylated CpGs are marked in red. (**d**) Genome-wide ratio of differentially up- and downregulated genes with concomitant change in methylation (≥ 3 CpGs). (**e**) Protein–protein-interaction network between the most interconnected differentially expressed and methylated genes. Points are scaled by the negative logarithmized FDR of differential expression and colored by log2 fold-changes. Edges are scaled by confidence of interaction. (**f**) Significantly correlated differential expression and enhancer methylation of CARD14, expression and TSS200 methylation of IRF8, expression and TSS200 methylation of CSNK2A2, and expression and TSS200 methylation of KRT17. Plots were generated using R v3.6.1^76^ software.

Elucidating the complex molecular mechanisms underlying UV-gene interaction might offer new insights into how UV modulates skin homeostasis and disease pathogenesis to help improve the prevention of UV-induced skin aging and related pathologies. In order to obtain a comprehensive picture of the molecular events regulating acute epidermal photobiology, 32 female Caucasian volunteers (Fitzpatrick phototypes 1–4) where irradiated with individually calibrated doses of 0.9 MED using a full spectrum solar simulator on three subsequent days on a sun-protected area on their lower backs. 24 h after the last irradiation, suction blister roofs were extracted from irradiated and control sites of each subject and gene expression profiling (Illumina RNA seq) and concomitant DNA methylation profiling (Illumina EPIC Arrays) were performed. Paired differential expression and methylation analyses between irradiated and control areas revealed that in total 20.5% (FDR < 0.05) of all interrogated CpGs and 32.4% (FDR < 0.05) of all detected gene transcripts were significantly altered in response to irradiation. These considerable changes were spread over the whole genome, with notable exceptions only occurring in the regions around the centromeres and in some constitutively heterochromatic regions e.g. on chromosome 13 (Fig. 1a). In general, a tendency towards hypomethylation was detected with 65.1% of all significant CpGs decreasing in methylation. Notably, the tendency towards hypomethylation increased from open sea regions to CpG-islands (Fig. S1 a).

Large blocks of the genome have previously been shown to be hypomethylated in chronically sun-exposed epidermal samples in comparison to protected skin^17,21^ and have also been associated with clinical measures of photoaging^17^. How quickly this epigenetic imprinting in response to UV exposure occurs however, is so far unknown. We thus investigated whether early indications of photoaging were already detectable after acute repetitive UV irradiation and analyzed the methylation status within the previously reported regions^17^ in our data. We found that in over a fifth of the originally described genomic blocks (49/224) the observed methylation changes after acute irradiation correlated very well with the reported patterns (Fig. 1b), differing mainly in magnitude in comparison to chronically exposed skin. This delivers evidence that epigenetic alterations in response to extrinsic stimuli can manifest quickly after external stimulation and suggests that even few repetitive sunburns can be sufficient to impact epigenetic imprinting in genomic regions associated with extensive photoaging.

Considering the extent of alterations in methylation patterns in response to acute irradiation and the universal role of DNA methylation in cancer biology, we then also performed a comparison of pan-cancer methylation signatures^22^ to our data, to establish if any overlap in signatures could be observed. The analysis revealed a small number of genomic regions with methylation changes post-irradiation very much reminiscent of those found aberrantly methylated in cancerous tissue. Most of these showed extensive hypomethylation (Supplementary Fig. S2a,b). Whether these alterations are in fact linked to carcinogenesis or purely a product of stochasticity will remain to be determined, but the overlap and extent of correlation raises concern and might warrant further investigation.

### Genome-wide correlative analyses of gene expression and methylation reveal coordinated changes in known and novel players of the UV response

Methylation can lead to long-lasting activation or repression of gene transcription and analysis of simultaneously regulated genes on methylation and transcription level have been shown to yield higher prognostic values in several pathophysiological states^23,24^. We thus mapped the most stably differentially expressed genes and CpGs by genomic position and observed a high proportion of genes with equally pronounced methylation changes. Analysis revealed that 29.3% of all upregulated genes (FDR < 0.05 and log2FC_abs_ > 0.5) harbored at least three differentially methylated CpGs (FDR < 0.05 and log2FC_abs_ > 0.2), whereas for downregulated genes this number increased significantly further to 34.3% (Fig. 1c,d), with a high number of genes exhibiting inverse correlations to their methylation state. Stratifying the significant CpGs within these genes by regulatory regions revealed they were most frequently located in enhancers and more seldom in exon regions (Supplementary Fig. S1d). Analyzing known protein–protein-interactions between these differentially regulated and methylated genes (DEMGs) using STRING^25^ revealed a network of highly interconnected proteins surrounding ESR1, the estrogen receptor α, which was found downregulated following repetitive irradiation (Fig. 1e). Estrogen receptor α expression has been shown to be reduced following UV irradiation in vitro before^26^, and its activity has been linked to photoimmune suppression in animal studies. In mice, estrogen receptor antagonists were found to exacerbate immune suppressive action in a dose-dependent manner with estradiol treatment exerting protective effects respectively^27^, and the estrogenic compound equol protecting against irradiation-induced carcinogenesis^28^. Notably, the core network also involved the similarly downregulated retinoic × receptor α, previously linked to a functional vitamin A deficiency in the skin following UV irradiation^29^ and thereby contributing to photoaging.

We next performed genome wide correlation analyses of all annotated genes and interrogated CpGs to identify significant linear correlations between gene expression and methylation in annotated functional gene regions. Modeling gene expression as a function of mean methylation for all CpGs in potentially regulatory gene regions (enhancers, 1,500 bp and 200 bp upstream of the TSS as well as exon regions) revealed 2,267 significant associations after multiple testing correction. Again, most of these associations were found with alterations in methylation patterns in enhancer regions. Among these highly correlated differentially expressed and methylated genes we identified several known and previously described actors in the UV response, such as CYP24A1, BRCA2, NOTCH2, FOXO3 and GATA3. Examples also included the observed hypomethylation and upregulation of CSNK2A2, a catalytic subunit of Casein kinase II, a ubiquitous serine/threonine protein kinase involved with a manifold of cellular processes, such as cell cycle control and apoptosis and the immune-modulatory keratin KRT17 (Fig. 1f), both of which have previously been associated with UV response and tumorigenesis. Remarkably some of the identified genes, e.g. CARD14 or IRF8 (Fig. 1f), have thus far not been associated with UV irradiation, possibly reflecting the variance between in in vivo and in vitro generated data. Interestingly however, CARD14 mutations have been observed previously in psoriasis patients. Gain-of-function CARD14 mutations in mice lead to spontaneous psoriasis-like skin inflammation by inducing activation of the IL-23-IL-17 axis in keratinocytes and thereby immune cell infiltration^30^. In contrast CARD14^−/−^ mice displayed attenuated skin inflammation in murine psoriasis models^31^. Demethylation-driven CARD14 activation in irradiated cells of the human epidermis might thus present a hitherto undiscovered mechanism of epidermal UV response. The transcription factor IRF8 was found concomitantly significantly hypermethylated and downregulated, which is significant given its function as a tumor suppressor and its frequent downregulation in various cancer types through epigenetic silencing^32–34^. Recently IRF8 has further been implicated in cutaneous wound healing^35^, the methylation-driven downregulation of IRF8 might therefore constitute a novel mechanism contributing to the observed impairment of wound healing following irradiation. Notably, IRF8 is located within one of the genomic regions differentially methylated in photoaged skin^17^, it would therefore be interesting to investigate its functional role in photoaging, even more so considering the age-associated impairment of wound healing in the skin and the increased risk of developing skin cancer that is associated with both chronic sun-exposure and higher age.

### Pathway analysis shows distinct functional enrichments for methylation-associated transcriptional alterations

**Figure 2:**
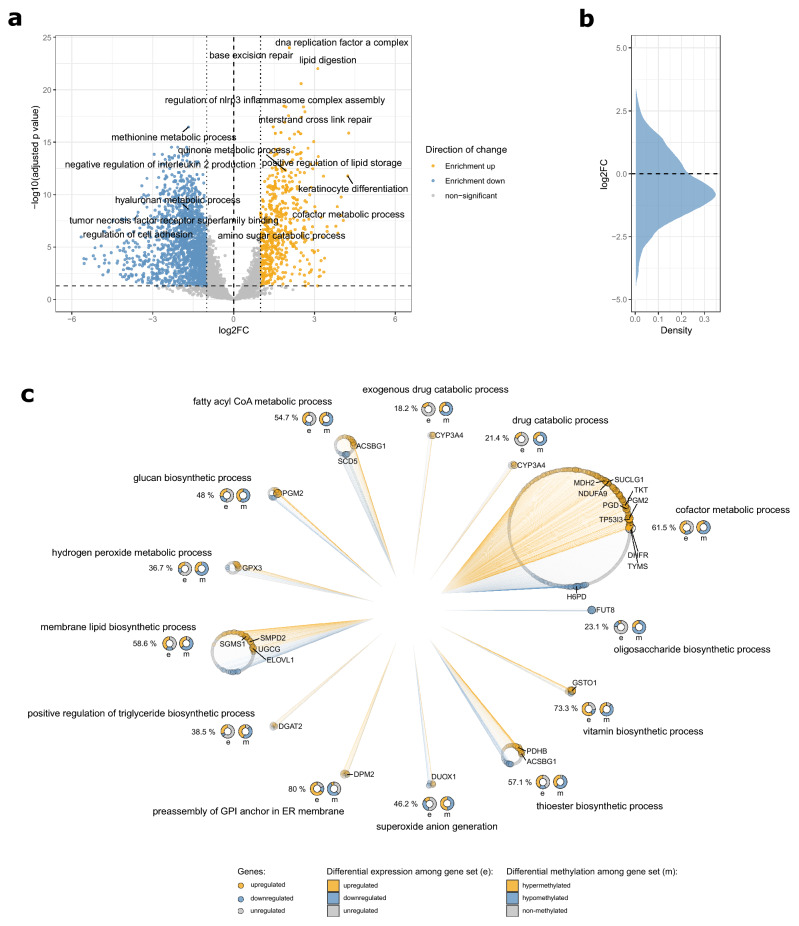
Biological pathways affected by simultaneous changes in both methylation and expression patterns in response to UV irradiation: (**a**) Volcano plot of enriched GO terms based on the analysis of differentially expressed genes with concomitant changes in methylation patterns (≥ 3 CpGs) with positively enriched pathways colored in yellow and negatively enriched pathways in blue (**b**) Distribution of log2 fold-changes in pathway enrichment after irradiation. (**c**) Enriched pathways involved with lipid biosynthesis and cofactor metabolic processes. Pathways are shown as circles with points corresponding to genes annotated to each respective pathway. Genes are colored by up- (yellow) or downregulation (blue) with size scaled to the negative log10 of the FDR derived from differential expression analysis and ordered by log2 fold-changes. Circles underneath pathway names represent proportions of differentially regulated (e) and methylated (m) genes within each gene set. Numbers to the left of the circles summarize the overall percentage of differentially expressed genes per pathway (FDR < 0.05). Plots were generated using R v3.6.1^76^ software.

Since the dissection of DEMGs revealed several genes which had previously not been connected to epidermal UV responses, we performed pathway analyses by means of gene set enrichment. Multiple pathways were strongly enriched with DEMGs, including DNA repair, immune signaling and stress response, strengthening the notion that DEMGs are at the heart of known and key response mechanisms to UV irradiation (Fig. 2a,b). In addition, a high number of enriched pathways were involved in metabolic processes, including some that had previously not been assigned to the canonical UV response pathways. Prominently these were linked to lipid biosynthetic and cofactor metabolic processes (Fig. 2c). Lipid synthesis in the epidermis is vital to skin permeability and barrier function, one of the skin’s most crucial functions. Outer epidermal keratinocytes secrete lamellar bodies, which are unique to the epidermis^36^ and contain phospholipids, glycosyl-ceramides, sphingomyelin, as well as cholesterol and numerous enzymes, including lipid hydrolases, such as β-glucocerebrosidase, acidic sphingomyelinase, secretory phospholipase A2 (sPLA2), and acidic/neutral lipases^37,38^. When the permeability barrier is perturbed, both the secretion and synthesis of lamellar bodies is stimulated, which allows for the rapid repair and normalization of permeability barrier function^39^. So far only few studies have evaluated the effect of UV on the stratum corneum. They provide evidence for an increased epidermal lipid synthesis in response to UV radiation and alterations of lipid profiles^40–42^, however these studies gave no functional correlation to genes or mechanisms involved. In addition, atopic dermatitis and psoriasis patients display modified lipid profiles and both groups are known to benefit from UV therapy. Induced DEMGs related to lipid biosynthetic processes might therefore provide evidence of an understudied UV response mechanism and potentially aid in identifying novel targets to help the regeneration of diseased skin. Differentially upregulated genes involved with other notably positively enriched pathways, such as TYMS and DHFR (Fig. 2c), are mainly involved in nucleotide synthesis and alterations to their increased expression may be part of important cellular responses that ensure proper DNA repair through the replenishment of DNA precursor molecules. The extent and magnitude of differential regulation in these pathways indicates high cellular priorities of these processes. These findings might warrant further investigation, as these pathways may be vital to maintaining genomic stability after UV irradiation.

### Molecular data allow precise inter-individual prediction of UV tolerance without experimental irradiation

**Figure 3:**
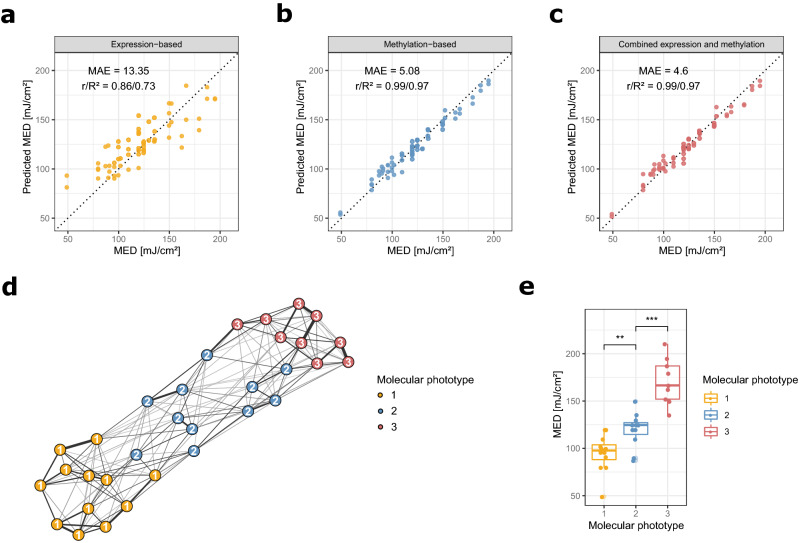
Prediction of inter-individual UV sensitivity from molecular data and identification of MED-correlated molecular phototypes: (**a**) Cross-validated predictions of MED from gene expression data using lasso regression models. (**b**) Cross-validated predictions of MED from DNA methylation data. (**c**) Cross-validated predictions of MED from combination of gene expression and DNA methylation data. (**d**) Fused similarity network generated from gene expression and DNA methylation data of irradiated samples, with nodes colored by molecular phototypes identified through spectral clustering. (**e**) Distribution of MED stratified by the molecular phototypes identified through spectral clustering on the fused similarity network. Statistical comparison was performed using unpaired two-sided t-tests. Plots were generated using R v3.6.1^76^ software.

Prediction of UV response is an important tool for risk assessment and prognostication of sun tolerance, photoaging, skin cancer and phototherapy. As a proxy for UV sensitivity, the Fitzpatrick scale is often used^7^. The Fitzpatrick scale or Fitzpatrick phototypes are a subjective, semi-quantitative scale made up of six phototypes that describe skin color by basal complexion, melanin level, and subjective assessment of inflammatory response to UV^43^. A more accurate way to measure UV tolerance is the experimental determination of the MED, which includes the acute irradiation of a test area with different UV dosages and a subsequent assessment of the minimal dose leading to erythema manifestation^13,44^. This method produces accurate and objective results, but is potentially harmful as it exposes the test subject to UV irradiation during the assessment. In the present study, subjects ranging from Fitzpatrick phototype 1 to 4 were analyzed and their MED assessed. As expected from previously published data^8,9,11^, stratification of donors using the Fitzpatrick classification was a relatively poor predictor of MED. For instance, the measured MED values for subjects of Fitzpatrick phototype 4 varied from 99.7 up to 210.4 mJ/cm^2^. We thus set out to explore if the assessment of individual UV sensitivity could be improved using molecular markers, forgoing the necessity to expose test subjects to harmful UV irradiation in the first place. We employed lasso regression models to attempt the prediction of individual UV sensitivity, as measured by MED, based on gene expression and DNA methylation data and a dataset combining expression and methylation features. The data included both irradiated and control samples, in order for the models to select features that would allow reliable estimation of UV tolerance irrespective of prior sun exposure of the tissue. The tenfold cross-validated predictions showed a high accuracy achieved by both expression- and methylation-based models (Fig. 3a,b), far outperforming the Fitzpatrick classifications with median absolute errors of 13.35 mJ/cm^2^ (expression-based) and 5.08 mJ/cm^2^ (methylation-based). Models built using DNA methylation features in particular were able to predict individual UV sensitivity to a remarkable degree, indicating a strong epigenetic component associated with UV tolerance. The combination of both expression and methylation data yielded the most accurate prediction model with a median absolute error of 4.6 mJ/cm^2^, suggesting further complementarity in the two data levels (Fig. 3c). Model performance was similar on irradiated and control samples, demonstrating the utility of the method irrespective of exposure status (Supplementary Fig. S3a–c). To our knowledge this is the first attempt to derive an accurate estimation of UV sensitivity purely from molecular data, which provides a reliable tool to assess individual UV tolerance, which importantly does not necessitate putting patients at risk of prior irradiation of the skin, as is the case with regular MED assessment.

### Multi-omics integration allows the identification of latent subgroups among irradiated samples

Considering the predictivity of the multi-omics data with regard to UV sensitivity, we decided to use an integrative approach to search for heterogeneity in the biological UV response. For this we integrated gene expression and methylation data from irradiated samples using similarity network fusion^45^. Similarity network fusion is a flexible network-based method for integrating different levels biological data, otherwise mostly employed in cancer research: First, a separate similarity network is created from each data level, with samples represented as nodes and similarities in profiles as edges. In a second step, the separate networks are then integrated using an iterative algorithm that strengthens edges between individual samples present in several levels of data, and finally converges into a fused similarity network that incorporates information from all the different data levels. In our case, this lead to a fused network incorporating information from both gene expression and DNA methylation data of the irradiated samples (Fig. 3d). Spectral clustering on the fused network then identified three latent subgroups in the multi-omics data, indicating differences in the biological responses to UV by different test subjects, and allowing their classification into distinct subtypes. The identified subtypes showed very high association to the MED (Fig. 3e) and allowed a better stratification of subjects based on UV sensitivity than Fitzpatrick phototypes, especially in the higher MED range (Supplementary Fig. S4a,b). Molecular subtyping of the skin with regards to UV response using these molecular phototypes (MPs) could prove helpful in developing preventive interventions, stratifying patients for risk factors (e.g. skin cancer and disease) and yielding deeper insights into molecular response mechanisms to irradiation.

### Molecular phototypes reveal divergent biological responses to UV irradiation connected to cytokine response, programmed cell death and DNA damage sensing and repair

To characterize the biological processes underpinning the variability of UV responses exhibited by the identified MPs, we assessed the importance of all pathways in the GO term collection with regard to UV response. For this we employed pathway-based machine-learning classifiers that were based on support vector machines using radial basis function kernels, capable of learning non-linear patterns from high-dimensional data. These classifiers were trained to predict irradiation status of a sample using gene expression data from a given pathway, and each “pathway model” was subsequently scored for how well it enabled discrimination between the groups in a repeated cross-validation scheme. This yielded a predictivity score for every gene set, ranking all pathways on a common scale whilst also capturing non-linear gene regulation patterns. Predictivity was assessed for all pathways stratified by the three identified MPs, allowing the identification of biological processes predictive for the UV response for a given subtype and thus also revealing pathways whose regulation diverges between the three subgroups. This resulted in a mapping of the whole pathway landscape with regard to UV response relevance within the three subtypes (Supplementary Fig. S5).

**Figure 4:**
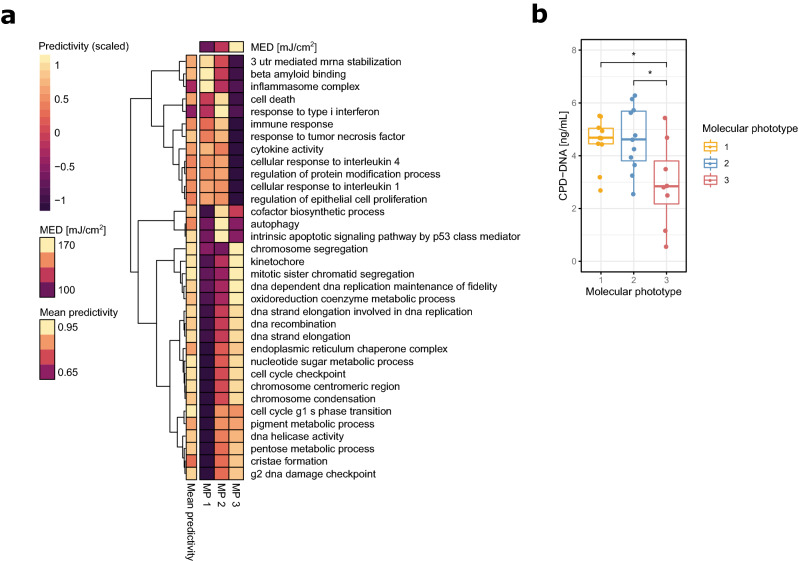
Molecular subtyping identifies heterogeneous biological responses to irradiation that correlate with innate UV sensitivity: (**a**) Heatmap showing the predictivity of the most defining pathways for each of the molecular phototypes to UV irradiation. The heatmap is scaled by pathway to enhance readability, average predictivity of a given pathway over all three molecular phototypes is shown to the left of the heatmap in original scale. (**b**) Extent of DNA damage in the form of cyclobutane pyrimidine dimers (CPDs) measured in the molecular phototypes 24 h after the last irradiation. Statistical comparison was performed using unpaired two-sided t-tests. Plots were generated using R v3.6.1^76^ software.

The on average most predictive pathways were involved with DNA damage response mechanisms such as cell cycle transition, DNA replication and chromosome condensation in concordance with the top pathways obtained using gene set enrichment earlier. Further analysis of the involved pathways however revealed divergent patterns between the three molecular subtypes (Fig. 4a). MP 1 and 2 for instance exhibited stronger signals in pathways associated with inflammatory and immune signaling in comparison to MP 3. In case of MP 1, the subgroup with the lowest average MED, these related strongly to inflammasome activation and cytokine response, both generally well-described responses in regards to UV irradiation in human skin^46–49^. In comparison, MP 2 exhibited decreased inflammasome predictivity scores but on the other hand a stronger type I interferon response than either MP 1 or MP 3. MP 2 was further singled out by stronger signals detected in apoptotic and autophagy pathways compared to the other subgroups. This might be connected to a stronger regulation in p53 related signaling pathways, as signaling by p53 class mediators showed increased predictivity in this subtype accordingly. Taken together this could indicate a higher efficiency in clearing cells with unrepairable DNA damage from the tissue. Both MP 2 and MP 3 further showed higher activities in pigment metabolic processes, which is in concordance with the stronger tanning responses observed in more UV tolerant skin^50^. MP 3 on the other hand, incorporating subjects with the highest recorded UV resilience in our cohort, was defined by the strongest pathway signals detected in cell cycle checkpoint and DNA synthesis pathways, as well as genes involved with chromosome condensation. These findings are indicative of a higher sensitivity of the DNA damage sensing machinery in MP 3 subjects in response to irradiation, which would provide a more tightly regulated cessation of DNA replication and thus more time for the repair of UV-induced DNA damage. This hypothesis led us to investigate the extent of DNA photodamage in the samples of study subjects from the different molecular phototypes. We profiled the most common and important form of UV-induced damage to the DNA, the formation of cyclobutane pyrimidine dimers (CPDs), a frequent cause of mutation in the skin after UV irradiation, that directly links UV damage to carcinogenesis^51^. Analysis of the extent of CPDs detectable in the samples revealed lower abundances of CPD-alterations in the DNA of MP 3 subjects compared to the other molecular phototypes (Fig. 4b). This supports not only the model predictions but also the hypothesis of a pigmentation-independent UV protective mechanism in highly UV tolerant skin after repetitive irradiation. The identification of the direct mechanics and the elucidation of key players involved with this response will be important directions for future studies, as they may have potential implications for skin cancer prevention.

## Discussion

Epigenetic changes are considered to play a fundamental role in establishing gene expression patterns and providing a genomic response mechanism towards extrinsic influences. However, the experimental evidence describing the extent of this response still remains somewhat limited in many biological processes. We have generated comprehensive methylation and expression profiling data to enable a more comprehensive examination of the intricacies of epidermal UV responses. Our results provide first hints towards an immediate acquisition of aging and cancer related epigenetic patterns in response to UV irradiation. In accordance with these findings, epidemiological studies have previously established a causal role for short term UV exposure (e.g. blistering sunburns) during childhood and adolescence in the late epidermal cancer pathogenesis^52,53^. The spectrum of driver mutations related to skin cancer provides unequivocal genomic evidence for a direct mutagenic role of UV light in carcinogenesis^54–56^. Meanwhile, genomic sites of mutation in skin cancer frequently coincide with CpG-islands^57,58^, regions of high DNA methylation density, which has been attributed to the higher vulnerability of 5-methylcytosine bases to CPD-formation^59,60^. Apart from potential mutagenic effects, recent publications also revealed that actinic keratosis samples already bear the classical methylation features of cutaneous squamous cell carcinomas^61^. These reports are consistent with the notion that epigenetic imprinting might present another common mechanism of both photoaging and carcinogenesis.

In general, substantial inter-individual variation in UV tolerability and cancer risk can be observed among Caucasian subjects. Genetic factors like polymorphisms of the melanocortin 1 receptor (MC1R) gene correlate with fairness of skin, UV sensitivity and enhanced cancer risk, however do not fully explain the diversity of UV responses, suggesting the possibility of epigenetic involvement in UV sensitivity and pathogenesis^44^. In support of this, the molecular data and methylation-based features in particular allowed the highly precise prediction of individual UV sensitivity without any prior irradiation, delivering new and strong evidence of an epigenetic component to individual UV tolerance. Further molecular evidence of the heterogeneity in response to irradiation was delivered by the molecular subtyping analysis, where clustering on the integrated multi-omics data revealed three different molecular phototypes (MPs) among irradiated samples with distinct biological signatures. The MPs differed most prominently in their association with pathways regarding cellular stress response, apoptosis/autophagy and DNA damage sensing and repair (MP 1–3, respectively). In an attempt to validate the predicted improved damage sensing and DNA repair of the most UV resilient MP 3, we analyzed the extent of cyclobutane pyrimidine dimers (CPDs) at 24 h after the last irradiation, as readout of UV-induced DNA damage and mutagenic potential. Significantly, this data indeed revealed a decreased amount of CPDs after UV irradiation in MP 3 compared to lower MPs. This is in line with some previous studies which showed lower CPD counts in irradiated samples derived from higher phototypes^62,63^, although no molecular mechanisms could so far be elucidated. Interestingly, studies of this type often suffered from substantial biological variation within each phototype as well, once more highlighting the need for better stratification and potentially explaining why the mechanisms leading to these observations could so far not be elucidated in ex vivo tissue. Notably, our study setup differed slightly from most of the previous by making use of a repetitive irradiation scheme, potentially widening the window for detecting inter-individual differences in response mechanisms.

The most intensely explored UV-protection mechanism of the human skin is melanin pigmentation. Melanin serves as a physical barrier that scatters UVR and as an absorbent filter that reduces the penetration of UV through the epidermis^64^. The efficacy of melanin as a sunscreen in darker skin is two- to four-fold higher compared to Caucasians^65^. However individuals with highly pigmented skin have been found 16–500 times less likely to present with skin cancer compared to individuals with fair skin^53,66–68^. The type of melanin produced also plays an important role in skin cancer risk determination. The photoprotective effects of melanin are mainly attributed to eumelanin. Pheomelanin on the other hand has only weak photoprotective properties and has even been found to contribute to carcinogenesis by a mechanism of oxidative damage^69^. Still, even less deeply pigmented ethnicities such as Asians present far lower skin cancer rates compared to Caucasians^53^, hinting towards the existence of additional cancer protective mechanisms apart from melanin. One possible explanation involves MC1R variants, which have been shown to confer an increased risk of melanoma and non-melanoma skin cancers, independently of skin pigment (including red hair phenotype)^70^. The increased expression of transcripts which are associated with nucleotide metabolism and DNA repair in our dataset might present another previously uncharacterized mechanism leading to higher cancer protection afforded by skin with high UV tolerance. The detailed characterization of these biological pathways and the analysis of their clinical significance will be important aspects for future studies.

Taken together, our analyses demonstrate the benefit of using multi-omics integration for elucidating complex and diverse responses by disentangling inter-individual variation caused by insufficiently precise subject groupings, such as Fitzpatrick phototypes. The presented data illuminates the diverse and interconnected impacts of repetitive UV irradiation on both transcriptomic and epigenetic patterning in the human skin and provides new insights on protective mechanisms of subjects with high innate UV resilience, that might have further-reaching implications for UV-induced carcinogenesis.

## Material and methods

### Recruiting

32 healthy female Caucasian subjects belonging to Fitzpatrick phototypes 1–4 were recruited, with twelve subjects belonging to phototype 1 + 2, ten to phototype 3 and ten to phototype 4. Subjects were aged between 30 and 65 years, with homogenous age distributions in each phototype group. Similar to previous studies^71^, exclusion criteria included tattoos or scars in the test area, pigmentation disorders, pregnancy and medication such as anti-histamines or anti-inflammatory drugs within two weeks prior to study start. A detailed listing of exclusion criteria can be found in the Supplementary information.

### Minimal erythema dose determination

Minimal erythema dose (MED) estimation is a quantitative method to report the amount of UV (particularly UVB) needed to induce sunburn in the skin 24–48 h after exposure, by determining erythema (redness) and edema (swelling) as endpoints. Individual MED was determined for every subject on the first day of the study following the protocols described in DIN EN ISO 24444^13^.

### Repetitive irradiation of test sites and sampling

The study sites were located in a sun-protected area on the subjects’ lower backs and were randomly split into control and test areas. On the second day of the study, the first irradiation of the test sites was performed using a SOL 500 full spectrum solar simulator (Hönle UV Technology). Intensities were chosen individually to reach 0.9 MED for all subjects or in other words 90% of the required minimal dose causing erythema in a given test subject. Irradiation to 0.9 MED was repeated in the same manner on the third day and once again on the fourth day of the study, leading to a cumulative irradiation of all test sites three times. On the fifth day of the study and 24 h after the last irradiation session of each subject, epidermal samples were taken using the suction blister method, as previously described^72^. For each subject, two suction blister roofs of 7 mm diameter were extracted from both control and test sites, one of each to be used to extract RNA for sequencing, the other to extract DNA for the DNA methylation analysis. This amounted to four suction blister roofs extracted per subject and a total of 128 samples.

### Nucleic acid extraction

Nucleic acid extraction was performed as previously described^71^. Tissue samples were suspended in the respective lysis buffers for DNA or RNA extraction and homogenized using an MM 301 bead mill (Retsch). DNA was then extracted using the QIAamp DNA Investigator Kit (Qiagen) according to manufacturer’s instructions. RNA was extracted using the RNeasy Fibrous Tissue Mini Kit (Qiagen) according to manufacturer’s instructions.

### Transcriptome sequencing

Transcriptome libraries were prepared using TruSeq Library Prep Kit (Illumina) and sequencing was performed at 1 × 50 bp on Illumina’s HiSeq system to a final sequencing depth of approximately 100 million reads per sample. Sequencing data was processed using a pipeline including Fastqc v0.11.7^73^ for quality control, Trimmomatic v0.36^74^ for quality based read trimming and Salmon v0.8.1^75^ for read mapping and quantification of transcript expression in the form of read counts and transcripts per million (TPM).

### Differential expression analysis

Differential gene expression analysis was performed based on the quantified read counts in R v3.6.1^76^ using DESeq2^77^. Linear models were fitted using a paired design matrix to account for inter-individual variation unrelated to the irradiation treatment. Genes were considered significantly differentially regulated with FDR < 0.05 after multiple testing adjustment by the Benjamini–Hochberg procedure.

### Array based methylation profiling

Methylation profiling was performed using Infinium MethylationEPIC arrays (Illumina)^76^. Methylation data was processed using the minfi package^78^ in R. Normalization was carried out using the functional normalization method^79^, which makes use of internal control probes present on the array to infer and correct for technical variation between arrays. Subsequent analyses used M values to describe CpG methylation levels, as their approximate homoscedasticity renders them superior for statistical testing compared to Beta values^80^.

### Differential methylation analysis

Differential CpG methylation analysis was performed in R using limma^81^. Linear models were fitted using a paired design matrix to account for inter-individual variation unrelated to the irradiation treatment. CpGs were considered significantly differentially methylated with FDR < 0.05 after multiple testing adjustment by the Benjamini–Hochberg procedure. To compare DNA methylation patterns with those previously described in chronically sun-exposed skin and cancer, we used lists of the respective genomic regions and their methylation status in photoaged skin^17^ and different types of cancer^22^, which were available from the Supplementary information. The originally reported methylation changes within these regions were then compared to the average difference in methylation of all significantly differentially methylated CpGs (FDR < 0.05) annotated to the respective genomic regions in our data, allowing for a region-wise comparison of differential methylation.

### Gene expression and methylation overlap and correlation analysis

For the calculation of overlap between genes and CpGs, only differentially expressed genes with absolute log2 fold-changes above 0.5 with at least three differentially methylated CpGs with absolute log2 fold-changes above 0.2 were considered, in order to uncover the most reliably differentially expressed and methylated genes. Pearson’s correlation coefficients of gene-CpG pairs were calculated as the sum of all gene transcripts for a given gene correlated with the mean of all CpGs belonging to a functionally annotated group (i.e. all enhancer CpGs) annotated to a given gene. Annotations such enhancer status, location in transcription start sites or within exons were extracted from the official manifest files for the Infinium MethylationEPIC array provided by Illumina via their website. Significance was assessed using linear models in R, with p-values being adjusted for multiple testing using the Benjamini–Hochberg procedure.

### Protein–protein-interaction analysis

Information on protein–protein-interactions (PPI) retrieved from the STRING^25^ database, accessed through the STRINGdb package^25^ in R. PPI information was retrieved for all differentially expressed genes (FDR < 0.05) with absolute log2 fold-changes above 0.7 with at least three differentially methylated CpGs (FDR < 0.05) with absolute log2 fold-changes above 0.5. The interaction query was performed using the standard combined interaction score threshold of 400. The resulting network was refined using in R using igraph^82^ by retaining only the top 20% of the most reliable edges based on the combined interaction score, with nodes disconnected from the core network being trimmed in the process. The resulting PPI-network was then visualized using igraph^82^.

### GO term enrichment analysis

Enrichment analyses were performed using the z-score method^83^ as implemented in the GSVA R package^84^, applied to the log2 transformed TPMs. GO term gene sets^85^ were downloaded originated from the Molecular Signatures Database v6.2^86^ and included all three sub-ontologies: biological processes (BP), molecular functions (MF) and cellular compartments (CC).

### MED regression models

Lasso regression models^87^ for the prediction of MED were built in R using the implementation provided in the glmnet package^88^ and interfaced using the machine learning framework mlr^89^. As lasso regression models perform automatic feature weighting by regularizing the absolute magnitude of coefficients, the models were trained on the full datasets, forgoing the necessity of prior feature selection. Furthermore, data from both control and irradiated samples was included in the training process, in order to allow accurate predictions irrespective of previous UV or sun exposure of a given sample. Model predictions and accuracy scores were extracted from tenfold cross-validation to avoid overfitting and derive unbiased predictions and estimates for the quality of model fit. Metrics used for judging model performance were median absolute error (MAE), as well as the Pearson correlation coefficient (r) and the coefficient of determination (R^2^).

### Similarity network fusion and clustering

After a filtering step, removing features which showed little correlation to MED and reducing feature matrices to 10% of their original size, gene expression (log2 transformed TPMs) and CpG methylation data (M values) from irradiated samples were integrated via similarity network fusion as previously described^45^ using parameter settings of $k = 10$(number of neighbors), $t=20$ (number of iterations) and $alpha=0.5$ (hyperparameter). Clustering on the fused network was then performed via spectral clustering as previously described^45^. Measures used for the selection of cluster numbers were the eigen-gap statistic and rotation cost as proposed in the original method description^45^.

### Pathway predictivity analysis

Pathway predictivity analysis was performed using GO term gene sets^85^ downloaded from the Molecular Signatures Database v6.2^86^. The pathway models were based on the support vector machine (SVM) implementation from the e1071 R package^90^, interfaced via the mlr^89^ machine learning framework. The models were trained by restricting the expression data (log2 transformed TPMs) to that of genes annotated within a given pathway and trained to predict sample irradiation status (control or irradiated to 0.9 MED) stratified by molecular phototype. The SVMs used the radial basis function kernel with hyperparameters set to $$gamma=\frac{1}{size of gene set}$$ and $$C=1$$. Accuracy of prediction was derived from 5 × 5-fold repeated cross-validation for each pathway model, giving insight on how well genes within the gene set allow a discrimination between UV irradiated and control samples while controlling for overfitting, and used as a measure of predictivity of the respective pathway to irradiation status.

### Profiling of cyclobutane pyrimidine dimers (CPDs)

CPD concentrations were determined using the OxiSelect UV-Induced DNA Damage ELISA Kit (Cell Biolabs) according to the manufacturer’s instructions.

### General data analysis and visualization

Data analysis in R further included the usage of the package data.table^91^ and dplyr^92^ for diverse data handling tasks, as well as the packages ggplot2^93^, ggpubr^94^, circlize^95^, and pheatmap^96^ for visualization. Mapping and annotation of gene identifiers was performed using the biomaRt^97^ and org.Hs.eg.db^98^ packages, utilizing the GRCh37 (hg19) human genome build.

### Ethics

The study was performed in agreement with the recommendations of the Declaration of Helsinki and all test subjects provided written, informed consent. Approval of the study protocol was granted by the Ethics Committee of the University of Freiburg (study code 016/1672).

## Supplementary information

Supplementary Figure S1.

Supplementary Figure S2.

Supplementary Figure S3.

Supplementary Figure S4.

Supplementary Figure S5.

Supplementary information.

## Data Availability

Data generated within this study has been deposited online at ArrayExpress, under the accessions E-MTAB-9251 and E-MTAB-9249.
