# Supplementary figures and images for: Concomitant DNA methylation and transcriptome signatures define epidermal responses to acute solar UV radiation
Source: Sci Rep. 2020 Jul 31;10:12918. doi: 10.1038/s41598-020-69683-8 (PMC7395768; doi:10.1038/s41598-020-69683-8)

**a**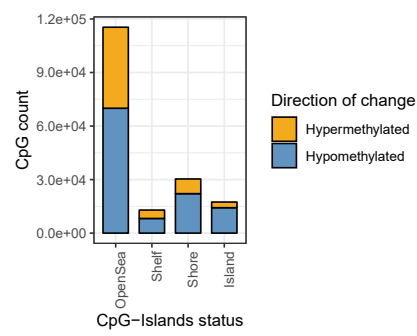**b**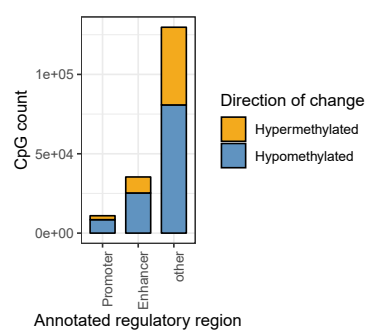**c**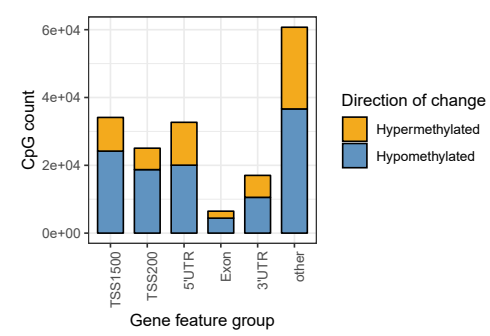**d**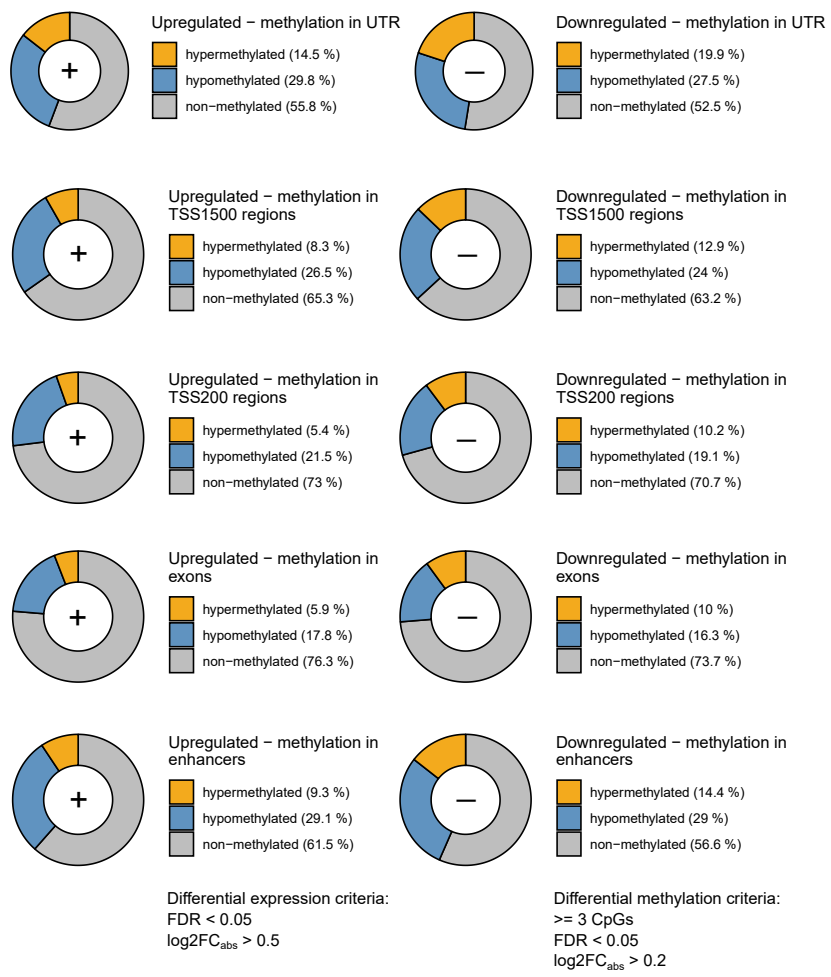

Supplement: Supplementary file 1 — Supplementary Figure S1. [file 41598_2020_69683_MOESM1_ESM.pdf]

**a**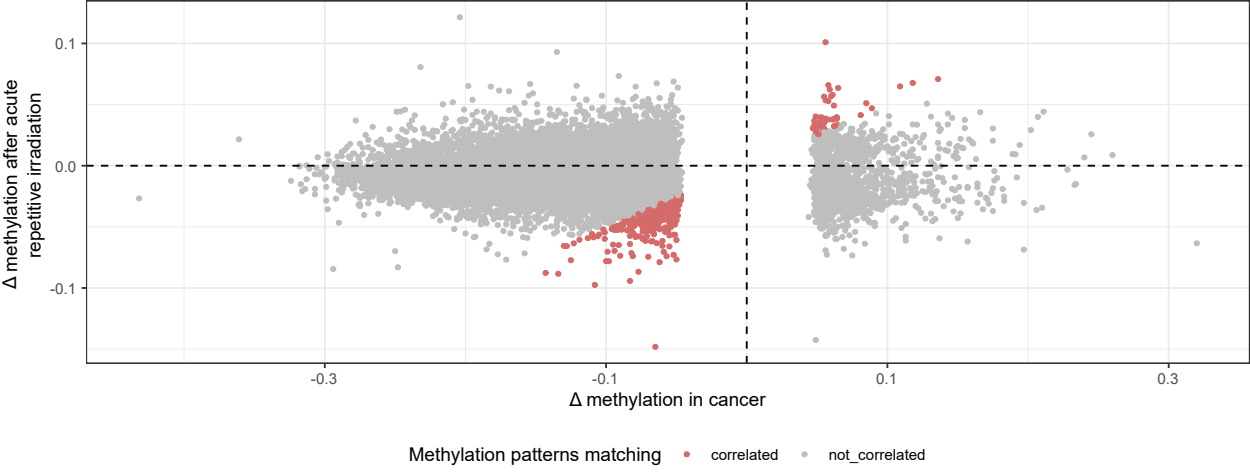**b**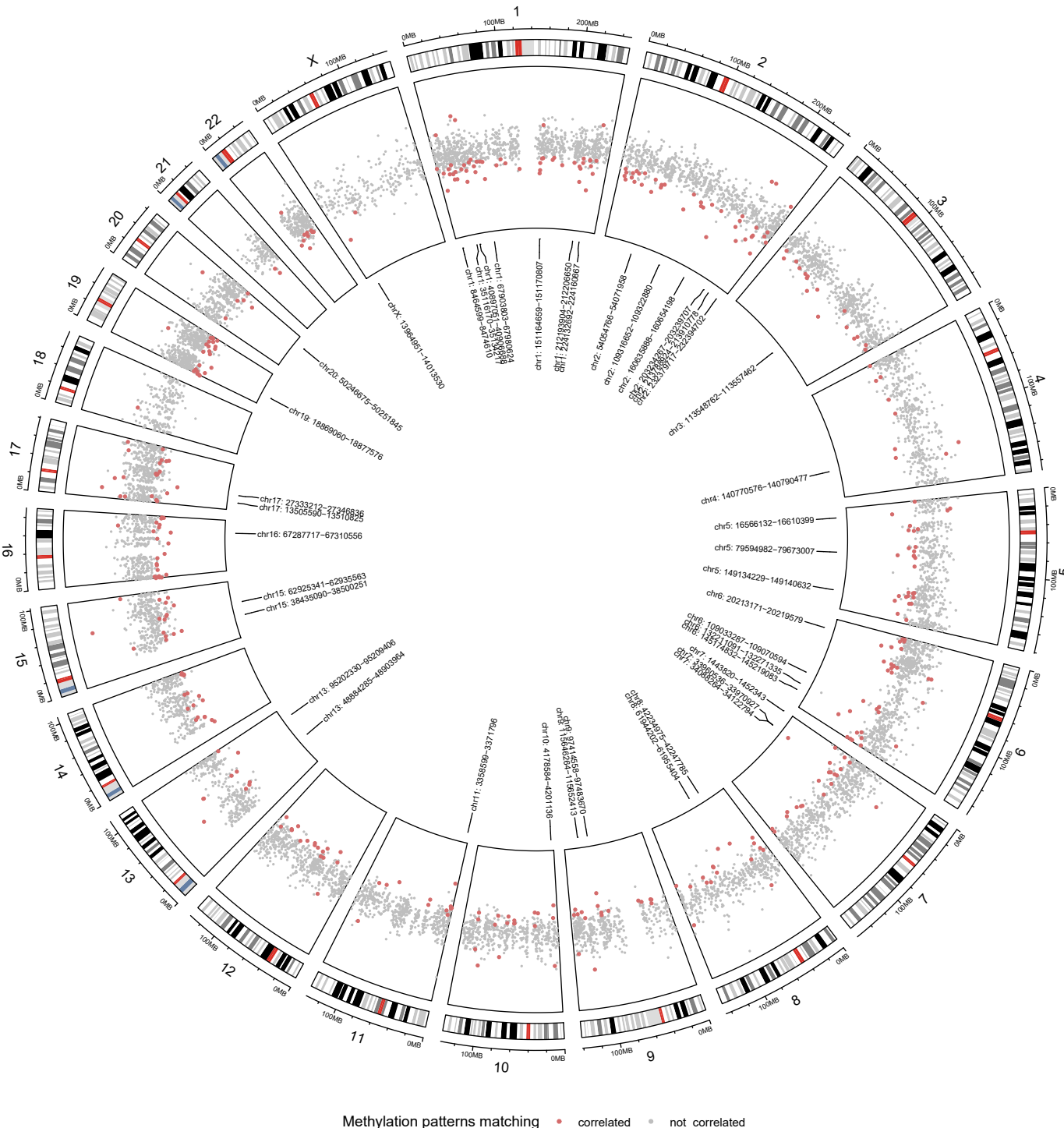

Supplement: Supplementary file 2 — Supplementary Figure S2. [file 41598_2020_69683_MOESM2_ESM.pdf]

**a**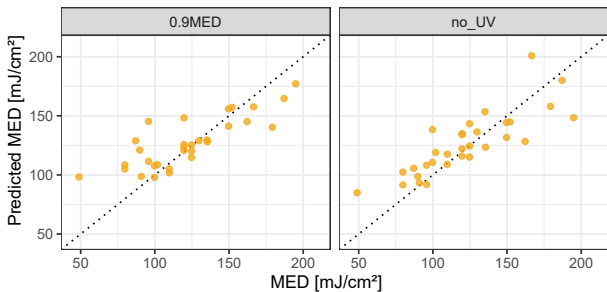**b**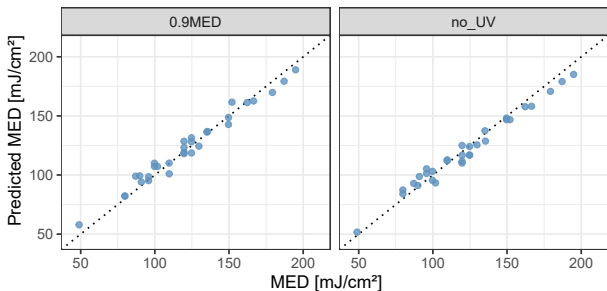**c**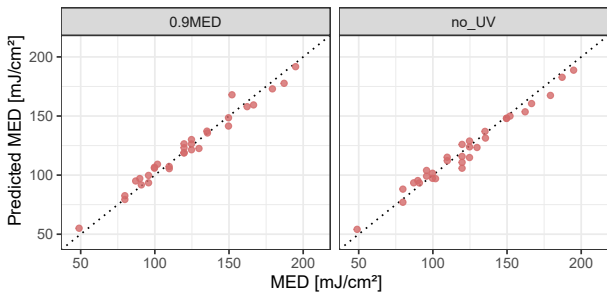

Supplement: Supplementary file 3 — Supplementary Figure S3. [file 41598_2020_69683_MOESM3_ESM.pdf]

**a**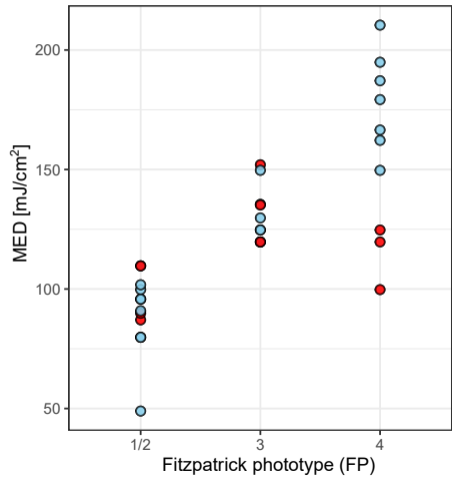**b**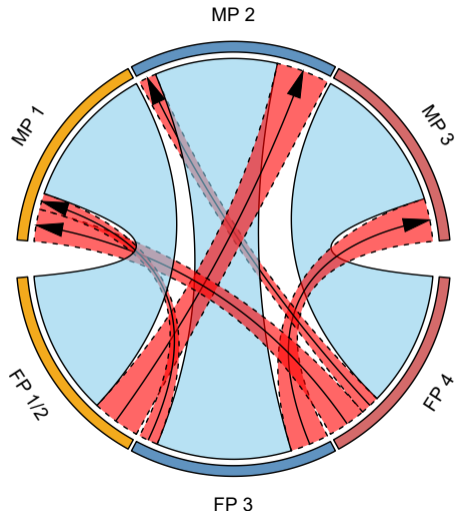

Supplement: Supplementary file 4 — Supplementary Figure S4. [file 41598_2020_69683_MOESM4_ESM.pdf]

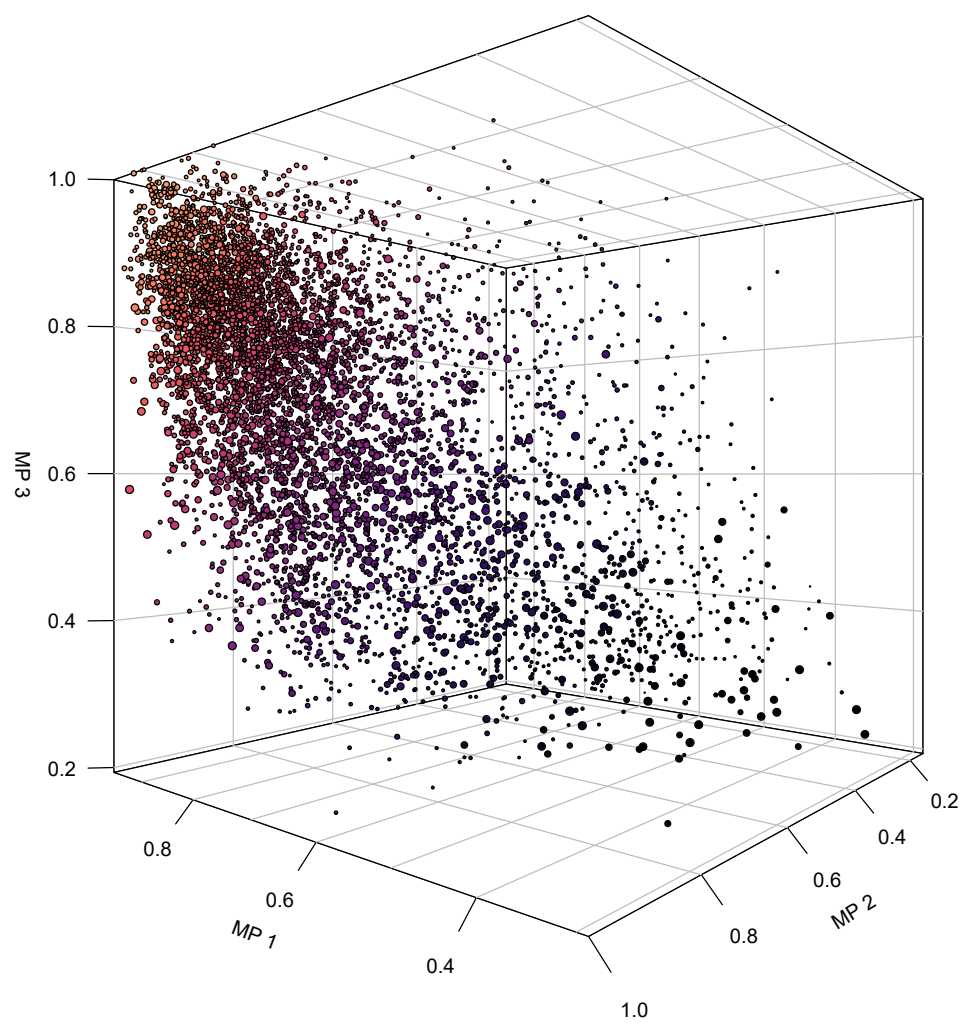

Supplement: Supplementary file 5 — Supplementary Figure S5. [file 41598_2020_69683_MOESM5_ESM.pdf]
