# Supplementary material for: Concomitant DNA methylation and transcriptome signatures define epidermal responses to acute solar UV radiation
Source: Sci Rep. 2020 Jul 31;10:12918. doi: 10.1038/s41598-020-69683-8 (PMC7395768; doi:10.1038/s41598-020-69683-8)
Supplement: Supplementary file 6 — Supplementary information. [file 41598_2020_69683_MOESM6_ESM.docx]

**Supplementary Material and Methods**

**Recruiting appendix – exclusion criteria**

- cancer in the past 10 years, skin cancer generally, as well as melanoma cases in family history
- systemic diseases such as diabetes, cardiovascular, liver, lung or kidney disease or chronic infectious diseases
- pregnancy
- BMI > 33
- smokers (> 10 cigarettes a day)
- acne, rosacea, psoriasis or atopic dermatitis
- scars or inflammation on test site
- sun bathing or solarium visits at least four weeks before study start
- usage of anti-histamine or corticoid-containing cremes at least four weeks before study start
- intake of anti-coagulants, immune-suppressive medication and retinoid-containing drugs at least two weeks before study start
- use of tanning and sun protection products at least one week before study start
- use of anti-bacterial, anti-allergic or similar cremes at least two weeks before study start
- cosmetic treatments at test site at least two weeks before study start
- acute infects with fever of at least 38.5 °C for at least 24 hours at least one week before study start
- sport, swimming or sauna at least 24 hours before study start
- allergies against ingredients of cosmetic products

**Supplementary Figure Legends**

Figure S1: **Epigenetic and correlated expression changes in response to irradiation:** (a) Differential methylation by CpG-island status. (b) Differential methylation by annotated regulatory regions. (c) Differential methylation by annotated gene features. (d) Ratio of significant hypo- and hypermethylation of CpGs in annotated regions of differentially expressed genes. Plots were generated using R v3.6.1^76^ software.

Figure S2: **Alterations of methylation patterns in genomic regions frequently associated with cancer in various tissues:** (a) Methylation in cancer vs healthy tissue against methylation in irradiated vs control samples for regions previously identified in various cancer tissues^23^. Colored in red are regions that show similar differential methylation after repeated solar simulated irradiation. (b) Genomic positions of the regions shown in (a). Colored bands in the karyogram mark centromeres (red) and heterochromatin status (grey to black). Plots were generated using R v3.6.1^76^ software.

Figure S3: **Accuracy of UV sensitivity prediction in irradiated and unirradiated samples**: (a) Cross‑validated predictions of MED from gene expression data using lasso regression models, split by sample irradiation status. (b) Cross‑validated predictions of MED from DNA methylation data, split by sample irradiation status. (c) Cross‑validated predictions of MED from combination of gene expression and DNA methylation data, split by sample irradiation status. Plots were generated using R v3.6.1^76^ software.

Figure S4: **Relationship between molecular and Fitzpatrick phototypes:** (a) Distribution of MED stratified by Fitzpatrick phototypes, with individuals reclassified into divergent molecular phototypes marked in red. (b) Chord diagram showing Fitzpatrick (FP) and molecular (MP) phototype classification of all subjects, with divergent assignments marked in red. Plots were generated using R v3.6.1^76^ software.

Figure S5: **Mapping of the pathway landscape in regards to predictivity for UV irradiation state:** Three-dimensional pathway predictivity map, with every point representing a gene set from the GO Term selection, showing the predictivity of each pathway for the UV response in molecular phototypes 1 to 3. Points are colored by average predictivity of each pathway over all three molecular phototypes. The plot was generated using R v3.6.1^76^ software.
